# Exploring functional and structural connectivity disruptions in spinocerebellar ataxia type 3: Insights from gradient analysis

**DOI:** 10.1111/cns.14842

**Published:** 2024-07-16

**Authors:** Xingang Wang, Hui Chen, Ru Wen, Peiling Ou, Yonghua Huang, Lihua Deng, Linfeng Shi, Wei Chen, Huafu Chen, Jian Wang, Changchun He, Chen Liu

**Affiliations:** ^1^ Department of Radiology, 7T Magnetic Resonance Translational Medicine Research Center, Southwest Hospital Army Medical University (Third Military Medical University) Chongqing China; ^2^ MR Research Collaboration Team Siemens Healthineers Ltd. Wuhan China; ^3^ Biomedical Engineering University of Electronic Science and Technology of China Chengdu China; ^4^ College of Blockchain Industry Chengdu University of Information Technology Chengdu China

**Keywords:** coupling, functional connectivity, gradient, spinocerebellar ataxia type 3, structural connectivity

## Abstract

**Aims:**

Spinocerebellar Ataxia Type 3 (SCA3) is a rare genetic ataxia that impacts the entire brain and is characterized as a neurodegenerative disorder affecting the neural network. This study explores how alterations in the functional hierarchy, connectivity, and structural changes within specific brain regions significantly contribute to the heterogeneity of symptom manifestations in patients with SCA3.

**Methods:**

We prospectively recruited 51 patients with SCA3 and 59 age‐and sex‐matched healthy controls. All participants underwent comprehensive multimodal neuroimaging and clinical assessments. In SCA3 patients, an innovative approach utilizing gradients in resting‐state functional connectivity (FC) was employed to examine atypical patterns of hierarchical processing topology from sensorimotor to supramodal regions in the cerebellum and cerebrum. Coupling analyses of abnormal FC and structural connectivity among regions of interest (ROIs) in the brain were also performed to characterize connectivity alterations. Additionally, relationships between quantitative ROI values and clinical variables were explored.

**Results:**

Patients with SCA3 exhibited either compression or expansion within the primary sensorimotor‐to‐supramodal gradient through four distinct calculation methods, along with disruptions in FC and structural connectivity coupling. A comprehensive correlation was identified between the altered gradients and the clinical manifestations observed in patients. Notably, altered fractional anisotropy values were not significantly correlated with clinical variables.

**Conclusion:**

Abnormal gradients and connectivity in the cerebellar and cerebral cortices in SCA3 patients may contribute to disrupted motor‐to‐supramodal functions. Moreover, these findings support the potential utility of FCG analysis as a biomarker for diagnosing SCA3 and assessing treatment efficacy.

## Graphical abstract

The experiment adhered to rigorous scientific methodologies and protocols, conducting the operational process with utmost attention to detail. FCG Values: functional connectivity gradient values; FC Values: functional connectivity values; FA Values: fractional anisotropy values; FC: functional connectivity; SC: structural connectivity. Four distinct gradient construction patterns and their corresponding inter‐group differential brain region were identified (A.B.C.D).

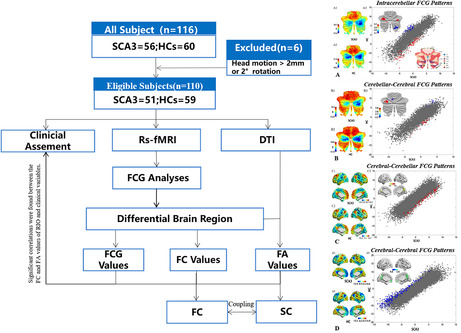

## 1 INTRODUCTION

Spinocerebellar ataxia type 3 (SCA3) is an autosomal dominant neurogenetic disorder characterized by an unstable CAG trinucleotide repeat sequence in the ATXN3 gene. Affected individuals exhibit an abnormal number of repeats, ranging from 52 – 86, which deviates significantly from the normal range of 12 – 44 repeats.^1^ SCA3 primarily impairs cerebellar function but also influences other nervous system regions, leading to a spectrum of non‐cerebellar symptoms that affect cognition, emotion, and sleep quality.^2^

Recent hypotheses increasingly suggest that alterations in neural networks contribute to the complex symptomatology observed in SCA3.^3^ The human brain features distinct functional hierarchies, with specific regions playing pivotal roles in executing complex cognitive functions, whereas others primarily support basic sensory and motor functions.^4^ Gradients are used to describe this hierarchical structure.^5^ Investigating the coordination among these regions and identifying disruptions that influence various neural systems could enhance our understanding of the complex symptoms associated with SCA3, potentially revealing biomarkers and therapeutic targets.

The hierarchical organization of the human brain is essential for integrating sensory and cognitive information.^6^ Disorders like SCA3, which impair connectivity and coordination among brain regions, can disrupt this organizational structure. Resting‐state (rs) brain functional networks can be decomposed into gradient components, revealing the connectome topography along a continuous spectrum. Our primary focus is on the first (i.e., principal) gradient, which accounts for the largest variance (29%). This level of explained variance aligns with recent findings from diffusion map embedding analyses of functional connectivity, and these results have been widely recognized.^5^, ^7^, ^8^ The organization spans from primary sensory networks to the transmodal default mode network, which integrates information across modalities and cognitive domains.^9^ Understanding these functional gradients may provide insights into how SCA3 impacts various cognitive processes.^7^

The relationship between functional connectivity (FC) and structural connectivity is a pivotal aspect of brain organization.^10^ Structural white matter connections are crucial in determining FC, and simulation studies suggest that functional dynamics contribute to shaping these structural connections through mechanisms of plasticity.^11^, ^12^ During normal brain development, the links between FC and structural connectivity strengthen as white matter connections and network dynamics converge, enhancing overall network efficiency and efficacy.^12^ However, in neurological disorders such as SCA3, FC and structural connectivity disruptions may occur, impairing neuronal function and integrity.^13^ Integrating data from FC and structural connectivity networks can improve the detection of subtle pathophysiological abnormalities with greater sensitivity than analyzing each modality separately. Despite advances, the underlying dynamics of structural and functional connectivity alterations in SCA3 remain poorly understood. Investigating these changes is critical, as the structure fundamentally underpins function. This comprehensive approach could yield valuable insights into the pathophysiology of SCA3 and facilitate the development of effective diagnostic and therapeutic strategies.

## 2 MATERIALS AND METHODS

### 2.1 Participants

From May 2017 to March 2022, our study prospectively enrolled 56 individuals diagnosed with SCA3 and 60 healthy controls (HCs), matched demographically. Peripheral blood was collected to determine the (CAG)_n repeat number in exon 10 of the ATXN3 gene coding sequence, using PCR and capillary gel electrophoresis; all participants were right‐handed. Exclusion criteria: Conventional MRI characteristics of all enrolled controls included the absence of known diseases and trauma history (e.g., leukoencephalopathy, tumors, vascular diseases, or mental illnesses), confirmed by two intermediate or above diagnostic doctors; history of alcohol or drug abuse, medication, or biofeedback therapy; severe claustrophobia or contraindications to MRI; head displacement >2 mm or rotation >2°. In total, five patients and one HC were excluded from the study. Our study has been registered in the Chinese Clinical Trial Registry (ChiCTR1800019901). All participants provided written informed consent for the study, and all procedures involving human participants were approved by the Ethics Committee of the First Affiliated Hospital of Army Medical University Review Board.

### 2.2 Clinical assessment

Neurological function was assessed using the Mini‐Mental State Examination and Montreal Cognitive Assessment (MoCA) to evaluate global cognitive function,^14^ the Digit Span (DS) test for verbal working memory,^15^ and the Rapid Verbal Retrieval (RVR) test for category fluency.^16^ Daily physical function was examined using the International Cooperative Ataxia Rating Scale (ICARS),^17^ Body Mass Index, the Scale for Assessment and Rating of Ataxia (SARA),^18^ and the Activities of Daily Living and Instrumental Activities of Daily Living scales.^19^ The Hamilton Rating Scale for Depression‐24 (HAMD‐24) was utilized to assess the severity of depressive symptoms.^20^ The ICARS and SARA scales were specifically applied to assess SCA3 patients. Together, these instruments measured various facets of neurological, cognitive, and emotional functioning among individuals with ataxia.

### 2.3 Data acquisition

MRI data were collected using a 3 T MAGNETOM Trio scanner (Siemens Healthcare, Erlangen, Germany). (rs‐fMRI) encompassed whole‐brain coverage, employing an echo‐planar imaging sequence with the following parameters: repetition time (TR) = 2000 ms, echo time (TE) = 30 ms, 36 slices, flip angle = 90°, slice thickness = 3.0 mm, field of view = 192 × 192 mm^2^, and voxel size = 3 × 3 × 3 mm^2^. For each participant, 240 volumes were captured over an fMRI scan duration of 8.08 min. High‐resolution T1‐weighted structural images were acquired using a gradient echo sequence, with parameters including TR = 1900 ms, TE = 2.52 ms, T1 = 900 ms, flip angle = 9°, 176 sagittal slices, matrix size = 256 × 256, field of view = 256 × 256 mm^2^, slice thickness = 1.0 mm, and voxel size = 1.0 × 1.0 × 1.0 mm^3^. DTI scans utilized a twice‐refocused diffusion‐weighted echo‐planar imaging sequence, featuring TR = 10,000 ms, TE = 92 ms, 65 slices, matrix size = 128 × 128, field of view = 256 × 256 mm^2^, acquisition voxel size = 2 × 2 × 2 mm^3^, 64 non‐collinear diffusion directions (uniformly distributed around a unit sphere *b* = 1000 s/mm^2^), and one image without diffusion weighting.

### 2.4 Diffusion tensor imaging (DTI) analysis

DTI data preprocessing was conducted using an established procedure with the FSL (FMRIB Software Library v6.0.2, http://www.fmrib.ox.ac.uk/fsl) and a diffusion toolkit. Initially, eddy current correction was performed to mitigate artifacts associated with directional variations in the magnetic resonance scanner's gradient fields or head motion.^21^ Subsequently, diffusion tensor models were generated for each voxel using a linear least‐squares fitting approach, utilizing the diffusion toolkit.^22^ For each participant, whole‐brain fiber tracking was executed in native space via the fiber assignment using the continuous tracking algorithm. The path termination was determined by fractional anisotropy (FA) values <0.2 or an angular threshold of 60°. To quantify the structural connectivity network nodes in native diffusion space, data were transformed from Montreal Neurological Institute (MNI) space to the individual‐participant diffusion space; high‐resolution functional partitions were then projected onto the individual‐participant diffusion space. Specifically, the structural image of each participant was co‐registered to their non‐diffusion‐weighted (*b* = 0) image in the native diffusion space using a linear transformation. The co‐registered structural images were subsequently mapped to the same stereotactic space as the customized template image, utilizing an affine transformation with 12 degrees of freedom and a series of nonlinear transformations generated from a set of 7 × 8 × 7 basis functions. The derived transformation parameters were inverted to transform these regions of interest from the functional partition to the native diffusion space.

### 2.5 Functional MRI analysis

rs‐fMRI images were processed with the DPABI package (v4.3.0, http://rfmri.org/DPARSF) in MATLAB 2017a (MathWorks).^23^ Data preprocessing included the removal of the first 10 volumes, slice‐timing correction, spatial realignment, and co‐registration based on the high‐resolution three‐dimensional anatomic volume. MNI space was normalized using a 12‐parameter affine linear transformation and nonlinear deformation. The images were resampled to 3 × 3 × 3 mm^3^,^24^ followed by wavelet despiking to address head motion artifacts. Additionally, regression of motion and non‐relevant signals (e.g., linear trend, Friston 24 head motion parameters, white matter, cerebrospinal fluid signal) and band‐pass filtering (0.01–0.1 Hz) were performed.

### 2.6 Functional connectivity gradient (FCG) analysis

Intracerebellar, cerebellar‐cerebral, cerebral‐cerebellar, and intracerebral FCGs were computed to delineate superimposed spatial relationships and continuous transitions between functional networks.^25^ For the intracerebellar FCG analysis, the resting‐state rs‐FC matrix for each participant was calculated between each pair of cerebellar voxels using Fisher Z‐transformed Pearson correlations. Subsequently, cosine distances were employed to create a matrix depicting the similarity among connectivity profiles. Diffusion map embedding was then utilized to extract a low‐dimensional embedding from the high‐dimensional connectivity matrix, a technique previously successful in exploring functional organization in studies of the cerebral cortex and cerebellum.^26^ Following the approach of prior studies, we set the manifold learning parameter (a) to 0.5.^9^ Diffusion embedding generated multiple continuous maps (gradients), which depicted the similarity of each voxel's functional connections within a continuous space. The first (i.e., principal) gradient captured the greatest data variability, representing the principal axis of macroscale functional organization in a particular brain structure as indexed by rs‐FC. Subsequent gradients, orthogonal to each other, captured progressively smaller proportions of data variability (e.g., second and third gradients). This gradient analysis was also applied to the cerebellar‐cerebral cortical, cerebral cortical‐cerebellar, and cerebral‐cerebral cortical FC matrices. The cerebellar‐cerebral cortical and cerebral cortical‐cerebellar gradients elucidated interactions between cerebellar and cerebral resting‐state networks, while cerebral‐cerebral cortical gradients focused on interactions within cerebral rs networks.

#### 2.6.1 Statistical analysis

In this study, gender differences between groups were assessed using the Chi‐square test. Additional demographic data were evaluated for normality. Non‐parametric equivalents were employed to analyze numerical data that did not conform to a normal/Gaussian distribution.

To identify altered gradient patterns, we compared gradient score differences between the patient group and HCusing a general linear model in SPM 12 software. Age, sex, and head motion were included as nuisance covariates. The significance threshold was established at a Gaussian random field (GRF)‐corrected *p* < 0.05 (voxel‐level *p* < 0.01, cluster‐level *p* < 0.05) using the Viewer program in DPABI. Furthermore, we calculated the FC between time series in brain regions exhibiting between‐group gradient differences and time series throughout the whole brain to pinpoint the sources of abnormal gradient patterns. A general linear model, adjusted for age, sex, and head motion, was employed to identify abnormal FC patterns. To investigate whether structural connectivity is compromised in regions where FC is abnormal, we used a generalized linear model to compare the FA values in these brain regions between the SCA3 group and the HC group, including age, gender, and head motion as covariates. Finally, Pearson correlation analyses were performed on quantitative values (gradient, FC, and FA) in brain regions of interest with between‐group differences and clinical scores for the SCA3 group. Adjustments were made for age, sex, education, and head motion.

**FIGURE 1:**
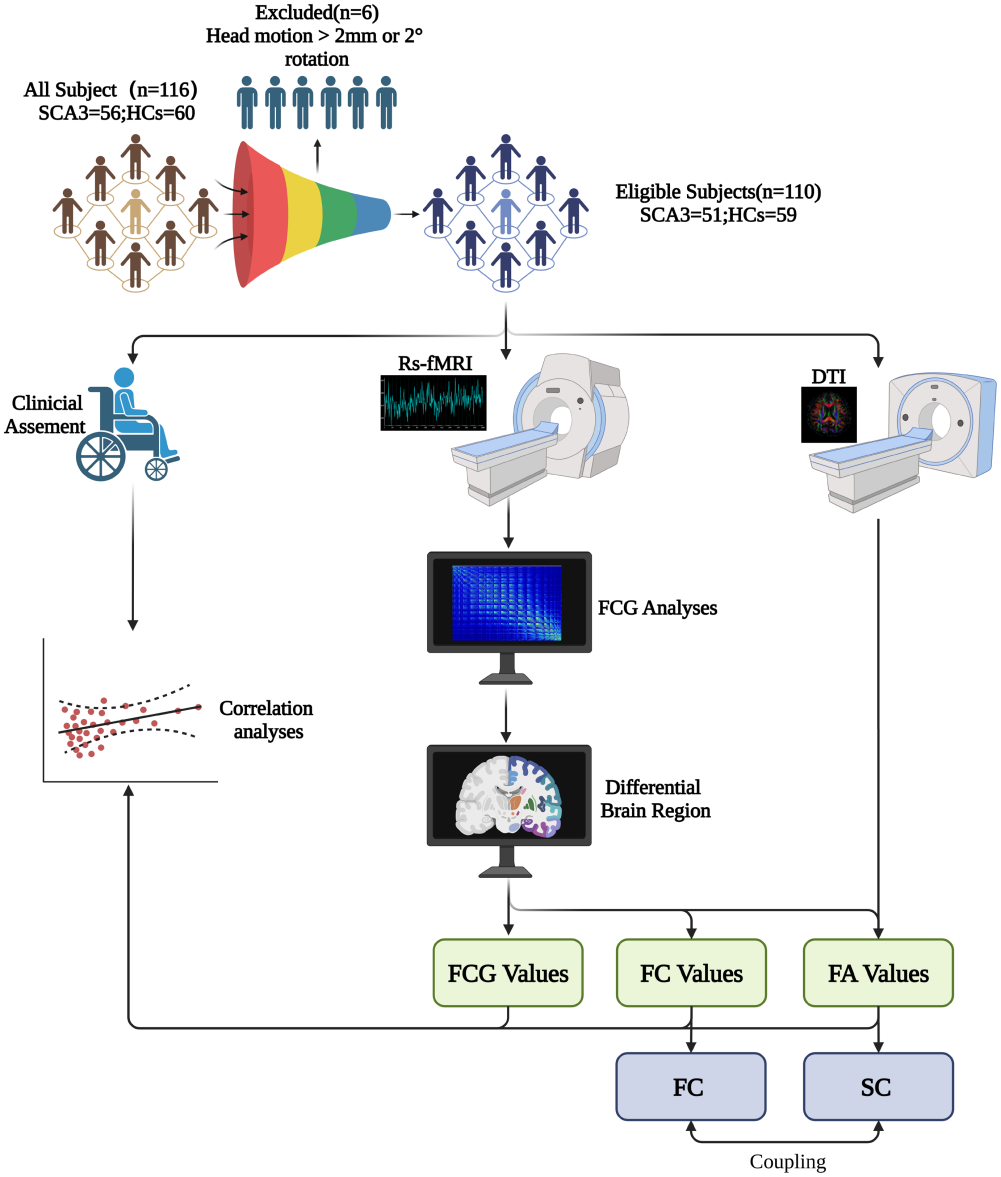
The experiment adhered to rigorous scientific methodologies and protocols, conducting the operational process with utmost attention to detail. FA Values, Fractional anisotropy values; FC Values, Functional connectivity values; FC, Functional connectivity; FCG Values, Functional connectivity gradient values; SC, Structural connectivity.

All experimental procedures were conducted following the protocols (see Figure 1).

## 3 RESULTS

### 3.1 Clinical Testing

In total, 51 SCA3 patients (19 women; age: 42.76 ± 12.50 years) and 59 age‐ and sex‐matched HCs (27 women, age: 42.64 ± 12.45 years) were included in the study. The CAG repeat range for the SCA3 group was 54–72 (the normal range was ≤44), which met the diagnostic criteria for SCA3. There was no evidence of a significant difference in age or sex between the two groups (*T* = 0.05, *p* = 0.96 and $x^{2}$=0.81, *p* = 0.37, respectively). Table 1 summarizes the participants' demographic, clinical, and cognitive variables. Significant group differences existed in all cognitive tasks except the DS task. Compared with HCs, SCA3 patients exhibited significantly worse performance (Table 1).

**TABLE 1:** Demographic, clinical, and cognitive variables.

Variable	SCA3 (*n* = 51)	HCs (*n* = 59)	T/W/x^2^ (*p* value)
Sex (M/F)	32/19	32/27	0.81 (0.37^b^)
Age (years)	18–70 (42.76 ± 12.50)	21–69 (42.64 ± 12.45)	0.05 (0.96^a^)
Onset age (years)	19–68 (37.62 ± 12.07)		
Disease duration (years)	1–21 (7.80 ± 4.10)		
CAG repeats	54–72 (65.37 ± 3.71)		
SARA	0–33 (9.93 ± 7.62)		
ICARS	0–75 (27.85 ± 18.87)		
BMI	15.06–31.25 (21.52 ± 3.18)		
HAMD	0–32 (6.65 ± 6.82)	0–17 (1.88 ± 3.25)	4.75 (0.00***^c^)
RVR	16–72 (35.90 ± 11.08)	25–80 (49.60 ± 11.05)	−5.77 (0.00***^b^)
DS	3–12 (8.41 ± 1.99)	5–16 (9.29 ± 2.10)	−2.16 (0.03^b^)
ADL + IADL	20–66 (28.12 ± 12.00)	20–21 (20.02 ± 0.13)	5.23 (0.00***^c^)
MoCA	10–30 (22.84 ± 4.95)	19–30 (26.97 ± 2.95)	−4.73 (0.00***^c^)
MMSE	13–30 (27.22 ± 3.34)	22–30 (28.76 ± 1.76)	−3.01 (0.00**^c^)

*Note:* Values are presented as ranges (means ± standard deviations).

Abbreviations: ADL, Activities of Daily Living; BMI, Body Mass Index; disease duration, time between onset and examination; DS, Digit Span; HAMD, Hamilton Rating Scale for Depression; HCs, health controls; IADL, Instrumental Activities of Daily Living; ICARS, International Cooperative Ataxia Rating Scale; M/F, Male/Female; MMSE, Mini‐Mental State Examination; MoCA, Montreal Cognitive Assessment; Onset age, age at onset of ataxia symptoms; RVR, Rapid Verbal Retrieval; SARA, Scale for the Assessment and Rating of Ataxia; SCA3, Spinocerebellar ataxia type 3.

^a^ Two‐tailed Pearson chi‐square test.

^b^ Two‐sample two‐tailed *t*‐test.

^c^ Mann–Whitney *U* test.

**p < 0.01; ***p < 0.001.

**FIGURE 2:**
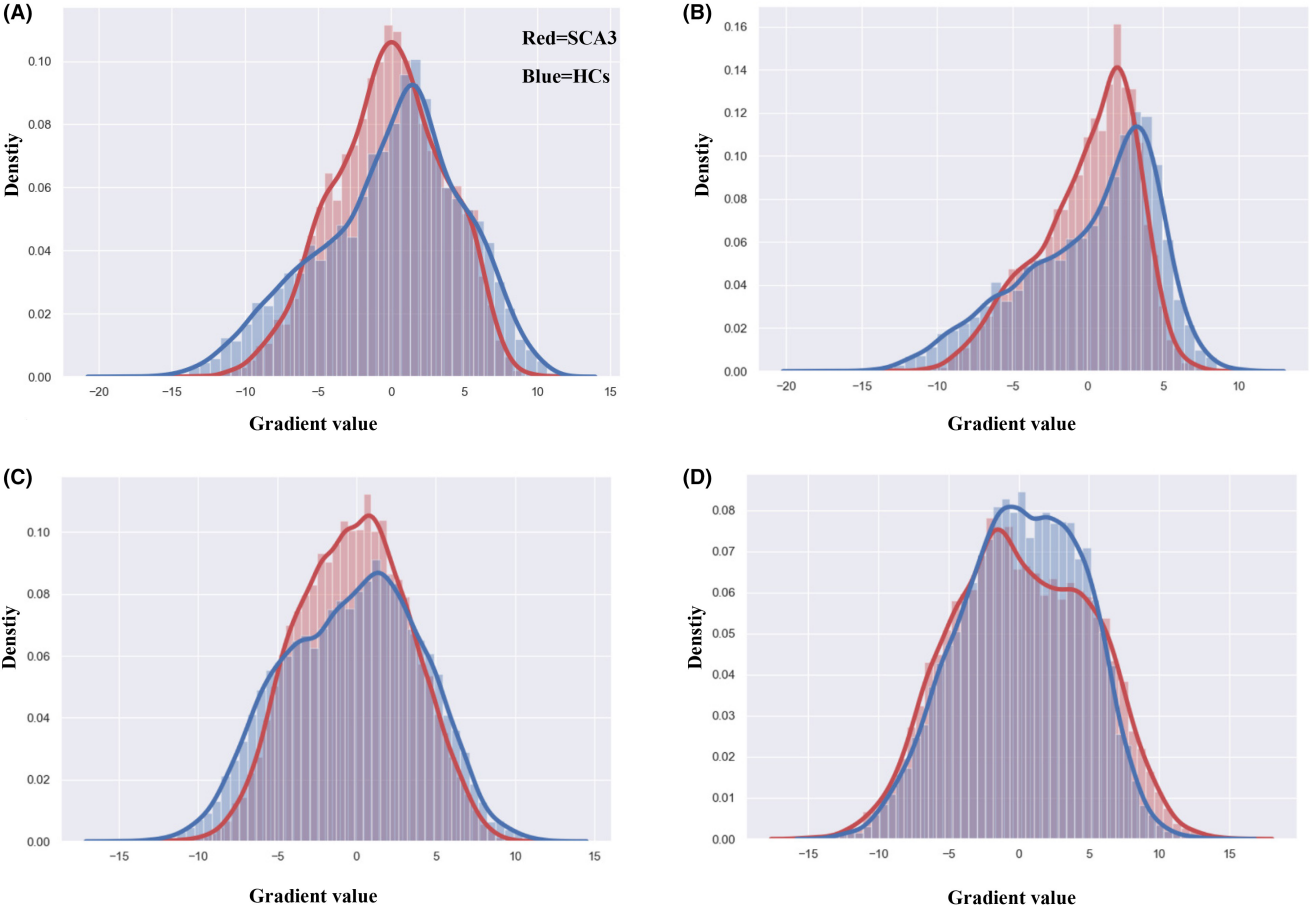
Compared to the HCs (health controls), compressed and expanded gradient patterns in SCA3 (spinocerebellar ataxia type 3), shown using density histograms. We observed significant compression in the lowest and highest parts of the principal gradient across the (A) intracerebellar functional gradient, (B) cerebellar‐cerebral cortical gradient, (C) cerebral cortical‐cerebellar gradient. (D) Expanded gradient patterns intracerebral functional gradient.

We conducted global histogram analyses to explore changes in the sensorimotor‐supramodal hierarchical gradient distribution among patients with SCA3. These analyses spanned intracerebellar, cerebellar‐cerebral cortical, and cerebral cortical‐cerebellar functional gradients. Results revealed a significant compression in the lower and upper segments of the principal functional gradient. This compression diminished the separation between the sensorimotor and supramodal extremes within each gradient. In contrast, the principal gradient within the intracerebral functional gradient displayed considerable expansion (see Figure 2).

### 3.2 Altered intracerebellar and cerebellar‐cerebral FCG patterns in SCA3 and the underlying abnormalities in FC and structural connectivity

**FIGURE 3:**
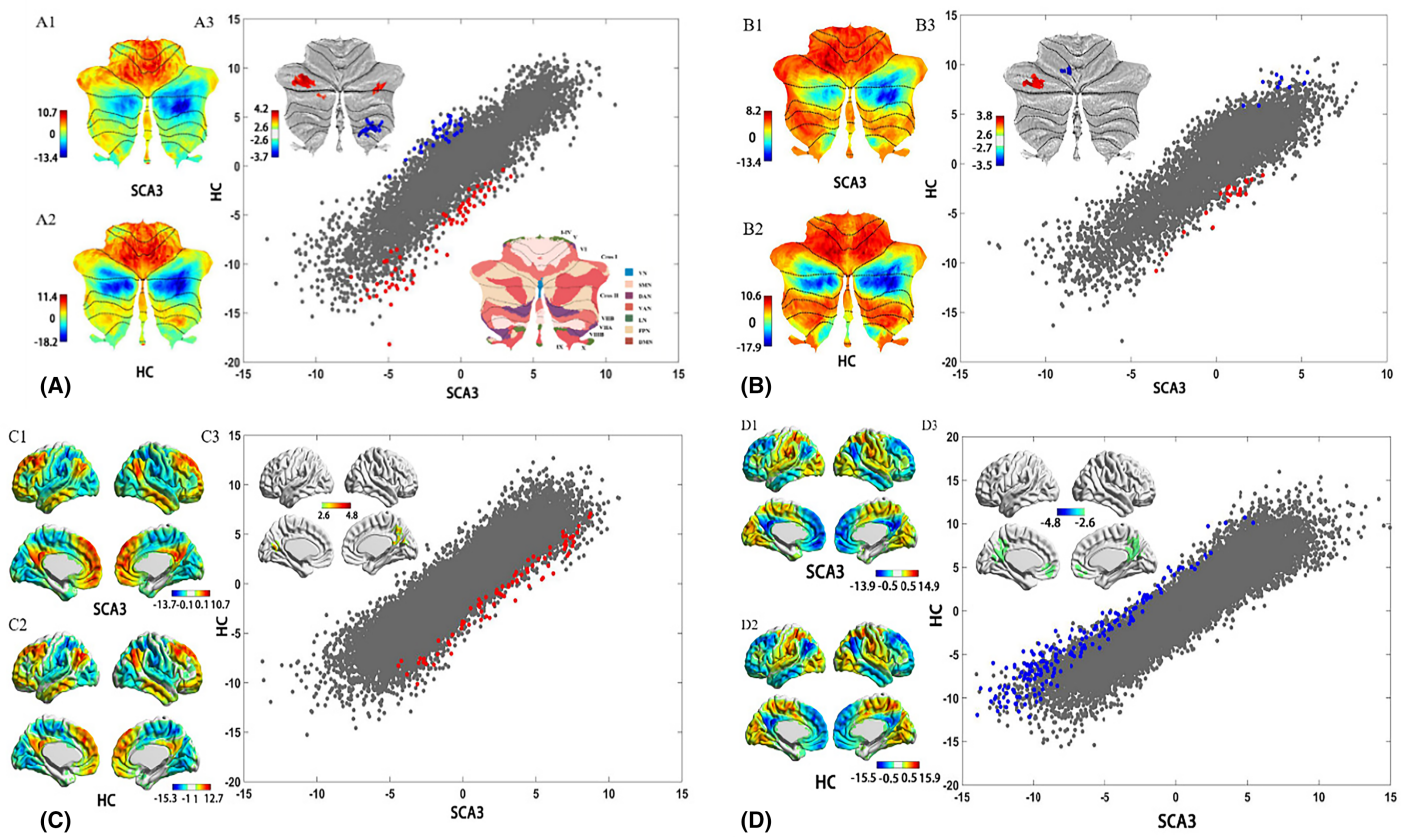
Group patterns and differences in principal gradient. (A) Cerebellar principal functional gradient calculated based on intra‐cerebellar functional connectivity. Scatterplot represents cerebellar gradient of SCA3 (x axis) versus cerebellar gradient of HC (y axis). Scatterplot colors correspond to group differences map as shown in top‐left corner of Figure 2 (A3): Higher gradient value in SCA3 (red), and lower gradient value in SCA3 (blue) compared to HC. (B) Cerebellar principal functional gradient calculated based on FC between cerebellum and cerebral cortex. (C) Cerebral principal functional gradient calculated based on FC between the cerebral cortex and cerebellum. (D) Cerebral principal functional gradient calculated based on intra‐ cerebral functional connectivity.

In patients with SCA3, specific spatial distributions of the principal functional gradient were observed within the cerebellum and between the cerebellum and the cerebral cortex (Figure 3A,B). Notably, gradient values in the right cerebellar lobule VII were significantly reduced, while those in the bilateral cerebellar crus I and left cerebellum crus II were significantly elevated, compared to HCs. Within the cerebellar‐cerebral gradient, values in the left cerebellar lobule VI were lower, whereas those in the left cerebellar crus I were higher among SCA3 patients (refer to Table 2). Significant alterations in FC and structural connectivity were also noted, particularly between the right cerebellar lobule VII and left cerebellar crus I in SCA3 patients (Table 2 and Figure S1A). Additionally, FC between the left cerebellar crus I and right cerebellar crus II was significantly reduced in SCA3 patients, although no changes in structural connectivity were observed (Table 3).

**TABLE 2:** Group differences in functional gradient values.

Brain regions	MNI coordinates			T value	Voxels (k)
	X	Y	Z		
Intracerebellar principal functional gradient					
Patients > Controls (FPN & DMN)					
Crus I_R	42	−78	−36	4.15	15
Crus II_L	−21	−90	−36	3.37	11
Crus I_L	−45	−69	−33	4.14	39
Patients < Controls (SMN & VAN)					
Lobule VII_R	21	−48	−51	−3.74	30
Cerebellar‐cerebral cortical principal functional gradient					
Patients > Controls					
Lobule VI_L (DMN)	−45	−69	−33	3.84	21
Patients < Controls					
Crus I_L (VN)	−12	−69	−15	−3.46	10
Cerebral cortical‐cerebellar principal functional gradient					
Patients > Controls					
Calcarine_R (VN)	4	−64	16	4.80	65
Precuneus_R (DMN)	4	−64	40	3.47	15
Intracerebral principal functional gradient					
Patients < Controls (DMN)					
Cingulum Mid_L	0	48	−16	−4.42	39
Rectus_L	−4	−40	52	−4.80	153

*Note*: Results (each type of gradient) are reported using a voxel‐wise GRF threshold of *p* < 0.05/3 and a cluster‐size threshold of *k* = 10.

Abbreviations: DMN, default mode network; FPN, frontal parietal network; L, left side of brain; R, right side of brain; SMN, sensorimotor network; VAN, ventral attention network; VN, visual network.

**TABLE 3:** Intergroup differences in functional connectivity.

Brain regions	MNI coordinates			*T* value	Voxels (*k*)
	X	Y	Z		
Intracerebellar functional connectivity					
Lobule VII_R	15	−66	−45	5.28	399
Crus II_L	−12	−90	−36	4.91	80
Cerebellar‐cerebral cortical functional connectivity					
Crus II_R	18	−90	−33	−4.22	85
Cerebral cortical‐cerebellar functional connectivity					
Calcarine_R	6	−21	42	−4.01	112
Intracerebral functional connectivity					
Lingual_L	−21	−78	−15	−4.92	87
Cuneus_R	9	−84	18	−5.10	166
Postcentral_L	−51	−15	30	−4.39	54
Postcentral_L	−15	−36	69	−5.53	131
Postcentral_L	−42	−24	57	−4.10	47
Postcentral_R	24	−42	63	−3.95	55

*Note*: Results (each type of gradient) are reported using a voxel‐wise GRF threshold of *p* < 0.05/3 and a cluster‐size threshold of *k* = 45.

Abbreviations: L, left side of brain; R, right side of brain.

### 3.3 Altered cerebral‐cerebellar FCG patterns in SCA3 and the underlying abnormalities in FC and structural connectivity

Regarding FC from the cerebral cortex to the cerebellum, SCA3 patients exhibited a unique spatial distribution in the principal functional gradient (Figure 3C). Compared with HCs, significantly increased gradient values were found in the right calcarine and right precuneus areas in SCA3 patients (Table 2). Furthermore, FC and structural connectivity between the right calcarine and right median cingulate cortex decreased (Table 3 and Figure S1B).

### 3.4 Altered cerebral‐cerebral FCG patterns in SCA3 and the underlying abnormalities in FC and structural connectivity

Regarding FC with the intracerebral cortex, the primary functional gradient exhibited a distinct spatial distribution in SCA3 patients (see Figure 3D). Relative to HCs, SCA3 patients demonstrated significantly reduced gradient values in the left rectus and median cingulate (Table 2). Additionally, reductions in FC were noted between the left median cingulate and the left lingual gyrus, as well as between the right cuneus and the left and right postcentral cortices. Corresponding declines in structural connectivity were also recorded (see Table 3 and Figure S1C).

### 3.5 Relationships among altered gradient values, FC values, FA values, and clinical variables

**FIGURE 4:**
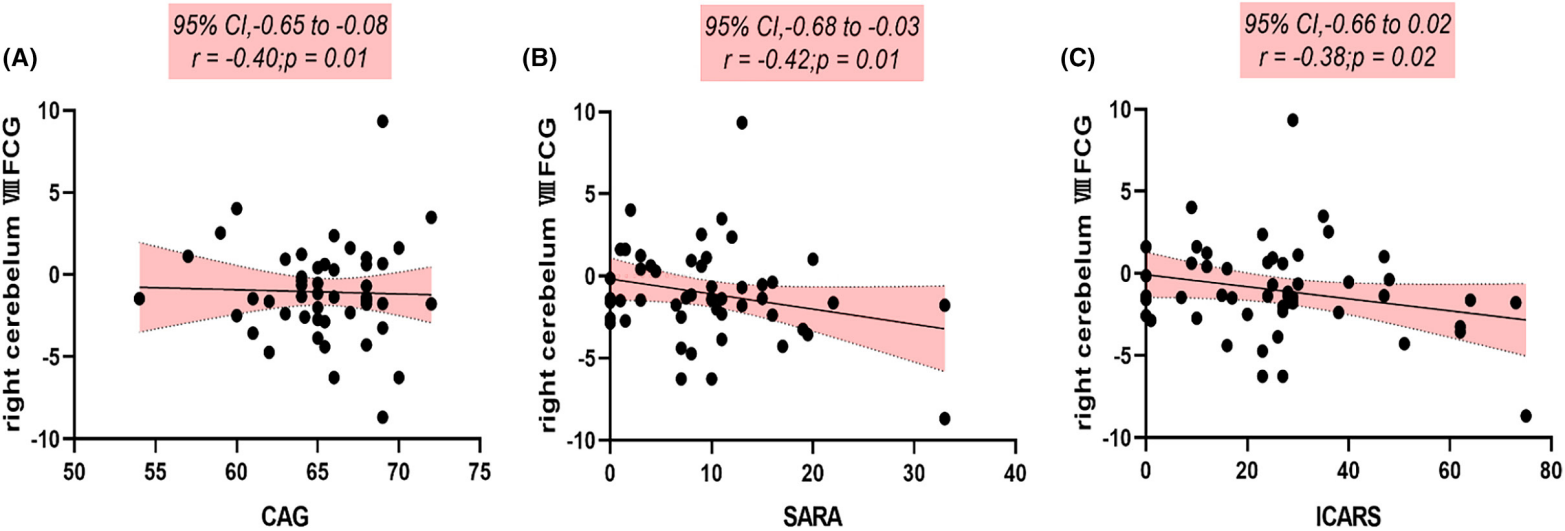
Correlation between gradient values and scales in abnormal brain regions of cerebral‐cerebellar. CAG, Cytosine, Adenine, Guanine cytosine; FCG, Functional connectivity gradient; ICARS, International Cooperative Ataxia Rating Scale; SARA, Scale for Assessment and Rating of Ataxia.

**FIGURE 5:**
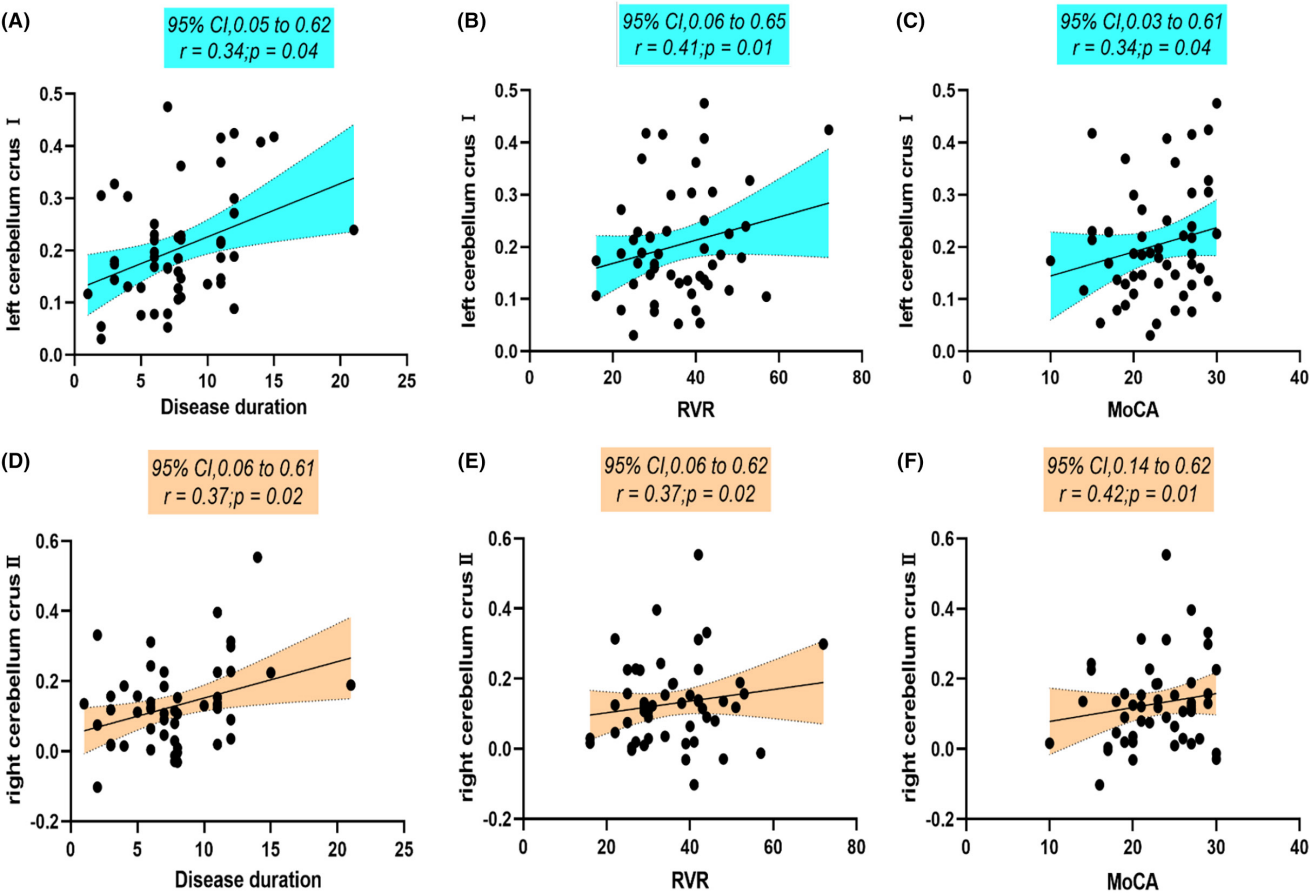
Correlation graph of functional connectivity scores and scale scores. (A–C) Correlation between FC values and scales in abnormal brain regions of intra‐cerebellar FC values, (D–F) cerebellar‐cerebral. MoCA, Montreal Cognitive Assessment; RVR, Rapid Verbal Retrieval.

Pearson correlation analyses were performed to investigate the associations between altered gradient values and clinical symptoms in SCA3 patients. These analyses revealed significant correlations between cerebral‐cerebellar FC gradient values in the right cerebellar lobule VIII and CAG repeats (*r* = −0.40, *p* = 0.01, GRF‐corrected), SARA score (*r* = −0.42, *p* = 0.01, GRF‐corrected), and ICARS score (*r* = −0.38, *p* = 0.02, GRF‐corrected) (refer to Figure 4). Further analyses explored the relationships between altered FC values and clinical symptoms in SCA3 patients. Notably, intracerebellar FC values in the left cerebellar crus I correlated with disease duration (*r* = 0.34, *p* = 0.04, GRF‐corrected), RVR score (*r* = 0.41, *p* = 0.01, GRF‐corrected), and MoCA score (*r* = 0.34, *p* = 0.04, GRF‐corrected) (Figures 5A–C). Additionally, cerebellar‐cerebral FC values in the right cerebellar crus II correlated with disease duration (*r* = 0.37, *p* = 0.02, GRF‐corrected), RVR score (*r* = 0.37, *p* = 0.02, GRF‐corrected), and MoCA score (*r* = 0.42, *p* = 0.01, GRF‐corrected) (see Figure 5D–F). However, no significant correlations were found between altered FA values and clinical variables.

## 4 DISCUSSION

This study identified abnormal functional hierarchy distributions within the cerebella and cerebra of SCA3 patients, alongside a decoupling of FC and structural connectivity. Notably, the gradients of sensorimotor and supramodal processing were compressed in SCA3 patients across intracerebellar, cerebellar‐cerebral cortical, and cerebral cortical‐cerebellar FC, resulting in decreased distances between sensorimotor and supramodal regions. This compression may indicate impaired simultaneity and interaction between low‐level and high‐level functional processing in SCA3 patients.^27^ Conversely, an expanded FC gradient within cerebral regions suggests increased distances between these regions. Concurrently, SCA3 patients demonstrated alterations in the correlation between FC and structural connectivity.

The principal gradient of cerebellar functional organization, represented by intracerebellar and cerebellar‐cerebral cortical modes, reflects a hierarchical structure transitioning from sensorimotor to cognitive processing.^28^ Employing two functional gradient construction modes, this study delineated the primary axis of cerebellar macroscale functional organization, uncovering a continuous spectrum of information processing within the cerebellum. Although both intracerebellar and cerebellar‐cerebral cortical functional gradients displayed a similar sensorimotor‐to‐supramodal gradient in HCs and SCA3 patients, significant alterations were observed within the cerebellum of SCA3 patients. These alterations included decreased values in the sensorimotor and ventral attention networks (SMN & VAN) and increased values in the frontoparietal and default mode networks (FPN & DMN). These findings are consistent with prior studies that report cerebellar cortical circuit disruptions and abnormal synchronization in schizophrenia patients.^29^ Furthermore, the intracerebellar FC gradient revealed pronounced abnormalities in supramodal cognition regions, specifically in the right crus I and left crus II, suggesting extensive cerebellar FC abnormalities in SCA3 patients. In parallel, the functional gradient within the cerebral cortex of SCA3 patients showed altered gradient values in the visual network (VN) and DMN regions. The establishment of cerebral‐cerebellar functional gradients exhibited increased values in VN and DMN regions, while cerebral‐cerebral gradients demonstrated decreased values in DMN regions. These aberrant patterns align with the characterization of SCA3 as a comprehensive brain disorder predominantly affecting the cerebellum, with consequent impacts on the organization of other brain regions.^30^

Compression of the functional gradient distribution within the cerebellar and cerebral cortices, particularly at the extremes associated with the SMN and task‐positive networks or the DMN, suggests impaired segregation between sensorimotor and supramodal cognitive systems in SCA3. This compressed architecture is likely due to increased connectivity within and between networks, leading to diminished network differentiation and reduced variability within each network. In conjunction with prior evidence of cerebellar abnormalities, these findings indicate that a less well‐defined global hierarchy characterizes the macroscale functional organization in SCA3.^31^, ^32^ Simultaneously, the functional gradient model within the cerebral cortex, as demonstrated intracerebrally, shows an expansion in contrast to the compression observed in cerebro‐cerebellar connections, suggesting a reduced capacity for internal coordination within the cerebral cortex. Thus, between intracerebral and cerebro‐cerebellar connections, the cerebral cortex's gradient construction exhibits distinct patterns. These results underscore abnormal hierarchical organization as a system‐level substrate of SCA3.

Focusing on gradient values in specific brain areas, particularly the right cerebellum lobule VIII, reveals significant relationships between genetic severity, symptom severity, and functional connectivity. Understanding these associations can enhance disease monitoring and inform targeted therapeutic strategies to preserve or improve connectivity in this crucial brain region.^33^ Altered FC values in specific regions were observed, including decreased FC in the left cerebellar crus I and the right cerebellar‐cerebral crus II. Positive correlations with disease duration, RVR score, and MoCA score suggest changes in internal functional integration and the cerebellum's role in regulating language and cognitive function.^34^, ^35^ These findings align with reports of similar functional impacts in the right cerebellar crus I/II in other neurological disorders.^36^, ^37^ Previous studies emphasized correlations between structural connectivity and FC.^38^ However, this study reveals mismatches under pathological conditions. Despite nearly uniform decreases in structural connectivity among SCA3 patients, FC exhibited decreased and increased values in specific regions, confirming the reorganization of brain networks in SCA3 patients and suggesting a separation between structural connectivity and FC, partially explaining the observed neurological and behavioral abnormalities in these patients.

The study has several limitations. The cross‐sectional nature of the data limits the ability to identify changes in brain FC and structural connectivity across different stages of SCA3. Although diverse preprocessing techniques were employed to mitigate interference, eliminating noise‐related changes in MRI signals was not achievable. Additionally, further whole‐brain quantitative analyses are necessary to explore the coupling between FC and structural connectivity, highlighting the need for more comprehensive research on decoupling DTI and fMRI to elucidate the structural and functional dissociation and its clinical implications in SCA3 patients. Given the rarity of SCA3 as a genetic brain disease, our patient recruitment numbers were constrained. Exploring gender differences within the patient group remains a limitation we plan to address in future research.

## 5 CONCLUSIONS

In summary, we investigated low‐dimensional representations of FC in SCA3 patients and HCs. Our findings revealed alterations in intracerebellar, cerebellar‐cerebral, cerebral‐cerebellar, and intracerebral connectivity among SCA3 patients. These alterations were correlated with diagnostic test scores, clinical symptoms, and cognitive impairment. Our findings extend beyond the existing motor‐centric models of SCA3 to encompass sensory and cognitive domains. The existing motor‐focused models of SCA3 include sensory and cognitive domains. These findings suggest that functional abnormalities in SCA3 originate from disruptions in the hierarchical organization of cerebellar and cerebral functional processing and the decoupling of FC and structural connectivity. Further research is needed to investigate these mechanisms in greater detail and to explore their potential as targets for therapeutic intervention. Our results provide a new perspective for understanding whole‐brain hierarchical relationships in SCA3 patients and exploring their physiological implications, along with new insights concerning neuropathological mechanisms in SCA3 patients.

## AUTHOR CONTRIBUTIONS

Chen Liu, Xingang Wang, Changchun He, Jian Wang, Hui Chen, and Ru Wen designed the study, analyzed the data, and wrote the main manuscript; Peiling Ou, Yonghua Huang, and Lihua Deng collected the data; Linfeng Shi and Wei Chen organized the data; Huafu Chen revised the manuscript. All authors contributed to the article and approved the submitted version.

## FUNDING INFORMATION

This work was supported by the National Natural Science Foundation of China: 81601478, 82071910; Senior Medical Talents Program of Chongqing for Young and Middle‐aged: 414Z395; Young and Middle‐aged Senior Medical Talents Studio of Chongqing; and Excellent Young Talent Fund of the First Affiliated Hospital of the Army Medical University: 2024YQBJ‐2.

## CONFLICT OF INTEREST STATEMENT

On behalf of all authors, the corresponding author states that there is no conflict of interest.

## TRIAL REGISTRATION INFORMATION

Chinese Clinical Trial Registry (ChiCTR):1800019901.

## PATIENT CONSENT STATEMENT

All participants provided written informed consent to participate in the study.

## Supporting information


Figure S1.

## Data Availability

The analyzed data in our study are subject to the following licenses/restrictions: The original anonymized imaging data analyzed in this manuscript will be used for research purposes. Requests for access to these datasets should be directly addressed to corresponding author.
