# Supplementary material for: Exploring functional and structural connectivity disruptions in spinocerebellar ataxia type 3: Insights from gradient analysis
Source: CNS Neurosci Ther. 2024 Jul 16;30(7):e14842. doi: 10.1111/cns.14842 (PMC11251871; doi:10.1111/cns.14842)
Supplement: Supplementary file 1 — Figure S1. [file CNS-30-e14842-s001.docx]

**Supplemental Data**

***Supplemental figure***


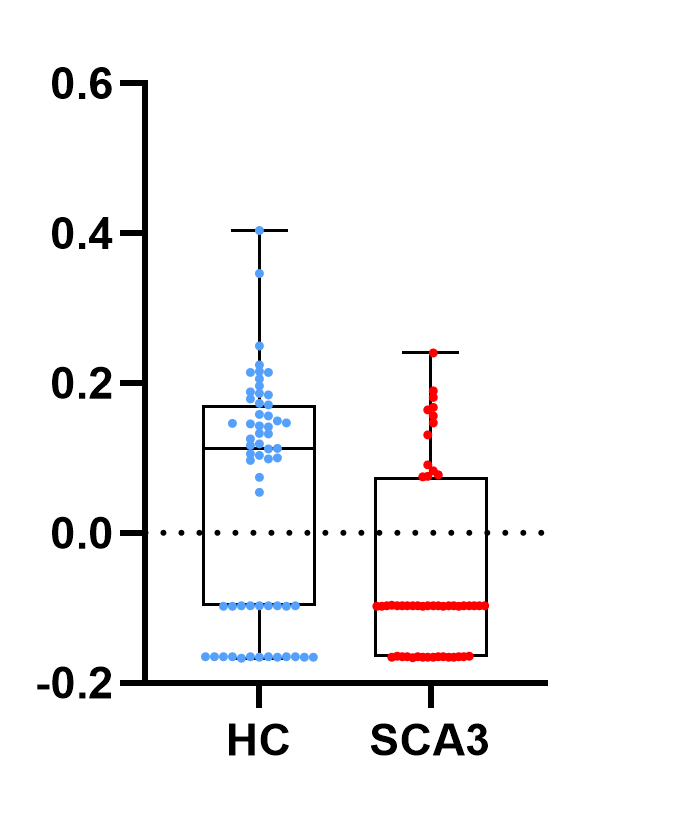


*95% CI,0.06 to 0.16*

*β = 0.11;p < 0.001*

**A**


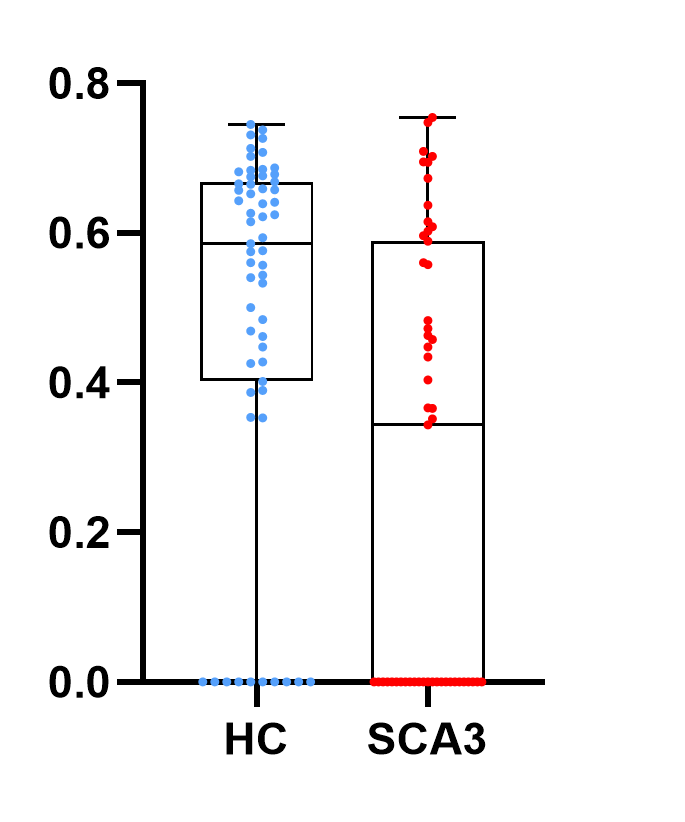


*95% CI,-0.01 to 0.11*

*β = 0.05;p = 0.09*

**B**


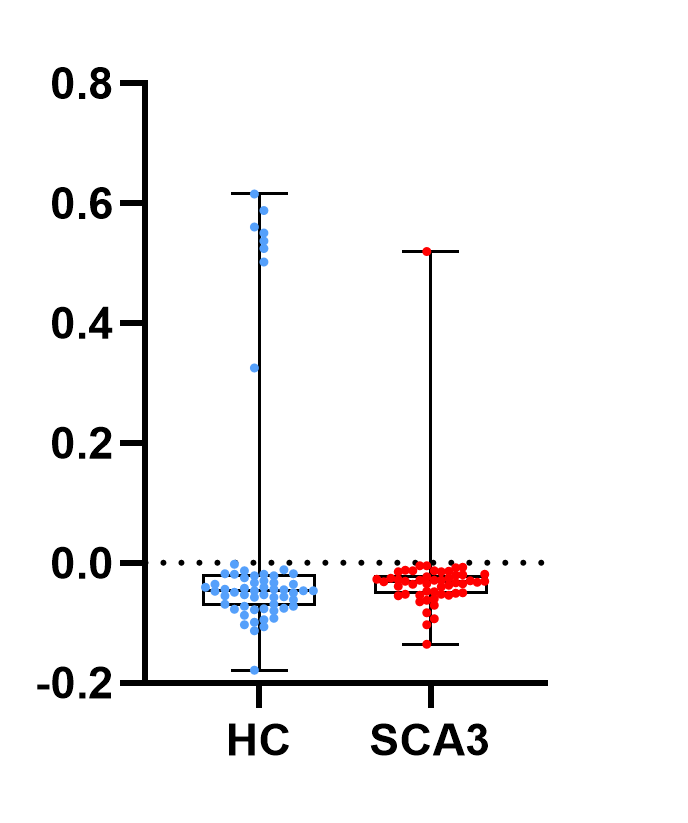


*95% CI,0.11 to 0.31*

*β = 0.21;p < 0.001*

**C**

FIGURE S1 Inter-group differences in structural connectivity. (A) Between the right cerebellum Ⅷ and the left cerebellum crus Ⅰ. (B) Between the right calcarine and the right median cingulate. (C) Between left median cingulate and the left lingual, the right cuneus the left and right postcentral cortex. HC: healthy control, SCA3: spinocerebellar ataxia type 3, CI: confidence interval. *β*: mean difference values.
